# Fractional diffusion models of cardiac electrical propagation: role of structural heterogeneity in dispersion of repolarization

**DOI:** 10.1098/rsif.2014.0352

**Published:** 2014-08-06

**Authors:** Alfonso Bueno-Orovio, David Kay, Vicente Grau, Blanca Rodriguez, Kevin Burrage

**Affiliations:** 1 Oxford Centre for Collaborative Applied Mathematics, University of Oxford, Oxford OX1 3LB, UK; 2 Department of Computer Science, University of Oxford, Oxford OX1 3QD, UK; 3 Department of Engineering Science, Institute of Biomedical Engineering, University of Oxford, Oxford OX3 7DQ, UK; 4 School of Mathematical Sciences, Queensland University of Technology, Brisbane, Queensland 4001, Australia

**Keywords:** fractional diffusion, Riesz potential, cardiac tissue, structural heterogeneity, electrotonic effects, dispersion of repolarization

## Abstract

Impulse propagation in biological tissues is known to be modulated by structural heterogeneity. In cardiac muscle, improved understanding on how this heterogeneity influences electrical spread is key to advancing our interpretation of dispersion of repolarization. We propose fractional diffusion models as a novel mathematical description of structurally heterogeneous excitable media, as a means of representing the modulation of the total electric field by the secondary electrical sources associated with tissue inhomogeneities. Our results, analysed against *in vivo* human recordings and experimental data of different animal species, indicate that structural heterogeneity underlies relevant characteristics of cardiac electrical propagation at tissue level. These include conduction effects on action potential (AP) morphology, the shortening of AP duration along the activation pathway and the progressive modulation by premature beats of spatial patterns of dispersion of repolarization. The proposed approach may also have important implications in other research fields involving excitable complex media.

## 1. Introduction

Excitable biological tissues, such as neural, cortical, gastric muscle or cardiac cells, are characterized by the generation and spread of timed electrical impulses that regulate their function, such as vision or contraction. The action potential (AP) represents changes over time in the electric potential of these cells that are the result of currents flowing across the membrane via the movement of ions. However, the extent to which electrical propagation is influenced by the highly complex, and heterogeneous nature of these tissues remains unclear. The spatial complexity of a medium can impose geometrical constraints on transport processes on all length scales that can fundamentally alter the laws of standard diffusion [1,2]. However, conventional modelling techniques represent these tissues as continuum media with spaced averaged properties, assuming a negligible contribution of their composite microstructure in modulating electrical conduction. In the particular case of cardiac muscle, and while many mechanistic findings have been obtained using these traditional approaches, their limitations to characterize tissue structure are well acknowledged [3]. New mathematical modelling techniques are thus needed to capture and explain the influence of tissue heterogeneity on cardiac wavefront propagation.

The fundamental modelling unit in understanding the propagation of electrical excitation is the cable equation. It describes the electrical propagation of an axial current along a thin fibre consisting of a homogeneous collection of excitable cells connected via gap junctions [4]. The model is constructed via an electrical circuit representation of a small path of the cellular membrane and the principle of homogenization to derive a continuous equation of the form
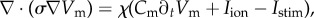
where *V*_m_ is the cellular transmembrane potential, and *I*_ion_ and *I*_stim_ the total transmembrane and stimulus currents, respectively. Model parameters are the tissue conductivity tensor, *σ*, the cell surface-to-volume ratio *χ* and the membrane capacitance, *C*_m_.

Through electric potential theory, it is known that an excitable membrane will induce electric fields through all components of the surrounding tissue [4]. This forms the basis for the bidomain model of cardiac electrophysiology
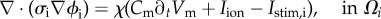
and
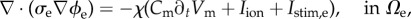
where the tissue is assumed to consist of two overlapping spaces: the intracellular, *Ω*_i_, and the extracellular, *Ω*_e_, domains, respectively, characterized by their corresponding conductivity tensors, *σ*_i_ and *σ*_e_. Electrical propagation is described by the scalar potentials in *Ω*_i_ and *Ω*_e_, *ϕ*_i_ and *ϕ*_e_, where *V*_m_ = *ϕ*_i_ − *ϕ*_e_.

**Figure 1.**
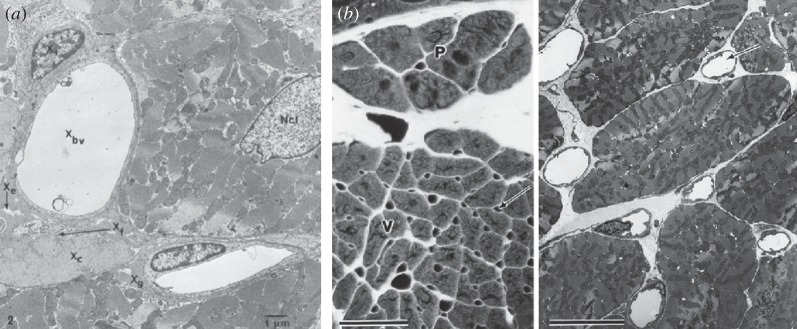
Transverse sections of cardiac muscle, illustrating multiple levels of structural heterogeneity. (*a*) Components of the extracellular space, including capillaries (*X*_bv_), empty space (*X*_e_), connective tissue (*X*_f_) and collagen (*X*_c_), embedded in ground substance (*X*_g_). Scale bar, 1 μm; *Ncl*, nucleus (7500×). Reproduced with permission from [5]. (*b*) Distribution of microvasculature in canine ventricular muscle (arrows), producing multiple indentations along the perimeter of each myocyte. The markedly heterogeneous distribution of interstitial space surrounding capillaries and myocytes is also appreciated. Left bar, 50 μm; right bar, 10 μm. Reproduced with permission from [6].

Discontinuities and heterogeneities in myocardial structure exist on several levels, as clearly evidenced by figure 1. Such a structural heterogeneity at different spatial scales may therefore pose possible limitations on both the monodomain and bidomain models as conclusive representations of cardiac tissue. First, the reasoning behind these models is that cardiac myocytes form collections of thin fibres that are arranged into sheet-like structures [3,5]. Gap junctions between the myocytes would preserve the cytosolic continuity, and so at a larger scale, this structure can be viewed from some aspects as a homogeneous domain. However, large differences in diffusion scales have been reported in the cytoplasm of mammalian cells [7], and gap junctions are known to have a much larger resistance compared with cytoplasm, which may be a source of discontinuous propagation on a local scale [8]. Furthermore, the brick wall structure of the myocyte sheets has a marked effect on propagation, and conduction delays depend on the number of adjacent cells connected to any given myocyte [9]. Thus, the argument for treating the intracellular domain as homogeneous is questionable at least, as also evidenced by the anomalous diffusion demonstrated in single particle-tracking experiments in cells, further supporting the high complexity of this medium [10–12].

In the case of the extracellular space, even more doubts can be raised. The extracellular domain is a complex mix of different tissue types, including fibrous tissue, blood vessels, collagen, fat and interstitial pores [5]. As a particular case in point, it is known that functional fibroblasts–myocytes coupling allows fibroblasts to transduce activity between otherwise unconnected myocytes [13]. Ephaptic coupling in the narrow extracellular regions between cells may also cause large changes in ionic concentrations that vary the electrical potential and can induce an electrical signal [14]. Additional factors such as the relative volumes of intracellular and extracellular space are also known to affect the resistance and distribution of cell-to-cell coupling [15].

Therefore, complex heterogeneous structures exist at a wide range of spatial scales in cardiac tissue. Under the abovementioned conditions, and even from a mathematical point of view, the applicability of a standard homogenization process to cardiac tissue can be questioned. In this type of settings, fractional (non-integer) models have been proposed as an alternative modelling framework (see appendix). Fractional spatial differential operators have been shown to incorporate the multi-scale effects of transport processes taking place in heterogeneous media. Applications include the filtration of solutes in porous soils [16], diffusion of water molecules in brain tissue [17], receptor-mediated transport of morphogens in developing tissues [18] or electrical charge transport in polymer networks [19]. Moreover, rigorous mathematical analysis on advanced homogenization techniques has established the connection between Brownian motions on disordered or complex structures and anomalous diffusion, as described by fractional diffusion models [20–22]. It is in this context of extended structures with spatially intricate patterns that fractional models can offer insights that traditional approaches do not offer. In particular, the structural characteristics of cardiac tissue suggest fractional diffusion as an appropriate modelling framework.

In this paper, we propose a family of fractional diffusion models to describe electrical propagation in heterogeneous excitable media, analysing their application to cardiac muscle as a representative case of composite biological tissue. More precisely, these models represent the modulation of the electrical field of a homogeneous conductor by the secondary electrical sources associated with its inhomogeneities (see §2). For the ease of presentation of these novel ideas, we concentrate on the case of isotropic conduction in a fractional monodomain formulation1.1
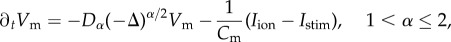
where *D_*α*_* is the diffusion coefficient and (−Δ)*^*α*^*^/2^ is the fractional Laplacian. These results can easily be extended to the anisotropic case by considering the fractional generalization of the standard diffusion operator, −(−∇ · (*σ*∇*V*_m_))*^*α*^*^/2^, so that, for *α* = 2, it recovers the standard monodomain formulation. The propagation model given by equation (1.1) for the transmembrane potential *V*_m_ is coupled to the system of ordinary differential equations1.2
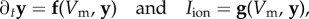
describing the cellular electrophysiological dynamics. Finally, given that *α* = 2 describes the perfectly homogeneous case (see §2), we focus our analysis on the upper part of the 1 < *α* ≤ 2 range, because we hypothesize this represents a tissue with a moderate-to-medium level of structural heterogeneity.

The outline of this paper is as follows. Section 2 presents the biophysical justification of our fractional diffusion description of cardiac tissue based on potential theory. Simulation results using our fractional models of electrical propagation are compared in §3 with *in vivo* human recordings and experimental data of different animal species. The agreement between simulations and experimental recordings offers novel insights into clinically relevant mechanisms of electrical wavefront propagation, namely conduction effects on myocardial depolarization, AP shortening along the pathway of activation and the modulated dispersion of repolarization. Hence, as discussed in §4, our results indicate the use of fractional diffusion models as a powerful tool to promote our current interpretation of the role of tissue inhomogeneities in modulating cardiac electrophysiology. The proposed approach may have, as well, important implications in unravelling the many facets of structural heterogeneity in other research fields where electrical propagation is highly influenced by complex media, such as soft muscle or neural tissue.

## 2. Biophysical justification of the fractional diffusion model

Both the monodomain and the bidomain formulations of the cable equation are well-accepted methodologies to describe the spread of electrical activity in excitable media [3]. The only difference between these modelling approaches and our proposed fractional diffusion models for heterogeneous excitable media is the replacement of the diffusive term (which describes tissue coupling) in equation (1.1) by the fractional Laplacian, (−Δ)*^*α*^*^/2^. None of the remaining terms is subjected to additional changes or affected by any spatial-dependence. Here, we aim to provide a biophysical interpretation for this new coupling term, which captures the degree of structural heterogeneity in the tissue.

In a statistical sense, the fractional diffusion process given by equation (1.1) without the reaction term can be viewed as describing the probability density function of an ensemble of particles undergoing a Lévy (jump) process, leading to a space–time scaling of the form *x* ∼ *t*^1/*α*^, that is, intermediate between normal and ballistic motion [1,2]. The closer *α* is to the value one, the more pronounced the heavy tailed distribution becomes, and the more likely that there is a huge range of spatial scales as to where these diffusing particles can lie. However, a further biophysical motivation is needed for fractional diffusion models in the context of excitable media. In order to make our justification beyond Lévy walks, we resort to potential theory. Consider a homogeneous domain in three-dimensional space with conductivity *σ* and a source *I* at point (*x*, *y*, *z*). Then, the electrical potential *ϕ* satisfies the solution of
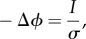
which at a field point (*x*′, *y*′, *z*′) is given by
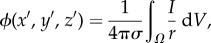
where *r* = [(*x* − *x*′)^2^ + (*y* − *y*′)^2^ + (*z* − *z*′)]^21/2^. Thus, in a homogeneous tissue, the electrical potential associated with a point source *I* = *I*_0_*δ*(*r*) (monopole) is given by2.1
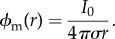
Equivalently, the electric potential associated with a dipole (two adjacent monopoles of equal and opposite sign, separated by a small distance *d*) is for 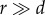
2.2
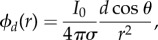
where *θ* is the polar angle between the dipole and the field point.

It is known that biological tissues give rise to volume conductors that are inhomogeneous in essence. This includes a variety of discontinuities in conductivity at multiple scales, from intracellular differences in diffusion, gap junctions connecting cardiomyocytes, to the presence of vasculature, fibrous, connective and adipose tissue or interstitial pores in the extracellular domain. As discussed in the classical textbook by Plonsey & Barr [4], continuity of the potential and the normal component of the current must be satisfied at the interface between regions of different conductivities. This corresponds to an equivalent double-layer source (see [4, §8.3.4]), which generates a field2.3
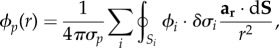
where *S_i_* denotes the *i*th surface on which a discontinuity in conductivity, *δσ*_i_, occurs. Here, **a_r_** is the unit radius vector from source to field and 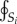 represents the integral over surface *S_i_*. The above equivalent source is considered a secondary source, because it arises only when a primary source has established a field and current flows across the interface separating the regions of different conductivities. Furthermore, and quoting the above-mentioned authors: ‘this view provides a conceptual (and possibly a computational) approach to considering the effect of inhomogeneities. In this approach, one finds the primary source field as if the volume conductor were uniform and infinite and then adds the fields generated by the secondary sources’.

Therefore, the total electrical field of a heterogeneous tissue can be approximated as the monopole component associated with a uniform conductor plus the perturbations that arise owing to tissue inhomogeneities
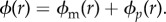


These secondary sources can actually be seen as a dipole modulation of the monopole given by equation (2.1), as by letting the *δσ*_i_ go to zero in equation (2.1), we recover the original monopole, but at the other extreme, we retrieve a dipole. This suggests a dependence on *r* ranging from 1/*r* to 1/*r*^2^. This insight allows us to make the connection to fractional models in terms of Riesz potential theory [23,24]. In 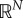, the fractional Laplacian can be written as
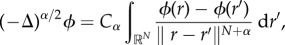


on a bounded domain with zero Dirichlet boundary conditions, whereas the case of reflecting boundary conditions can be also considered. Now, the solution of
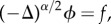


under the assumption that *f* is sufficiently regular and has compact support (so that it vanishes at infinity), can be written as2.4
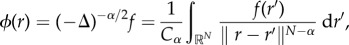
where 0 < *α* < *N* and2.5
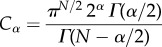
and *Γ*(*·*) denotes the Gamma function. So, for *N* = 3 and *f* = *I*_0_*δ*(*r*)/*σ*, then for *α* = 2, *C_*α*_* = 4*π* and *ϕ*(*r*) ∼ 1/*r*, which is consistent with the monopole described in equation (2.1). Equivalently, for *α* = 1, the dipole dependence *ϕ*(*r*) *∼* 1/*r*^2^ in equation (2.2) is recovered. Intermediate values 1 < *α* ≤ 2 can thus be interpreted as a smooth transition between these two types of electric potentials, representing a biological tissue with increasing degree of inhomogeneities as *α* approaches its ballistic lower limit. Note that this theory can be generalized to cope with anisotropy using the operator (−∇ · *σ*∇)*^*α*^*^/2^. Analogous continuity arguments have been used in other applications of fractional calculus to electrostatic theory [25,26]. Similarly, fractional models have been effectively applied to describe the presence of impurities in semiconductor heterostructures [27], where the generalization of fractal conductance, depending on restrain conditions in charge movement, has been also proposed [28,29].

It is important to recall that our interpretation of the fractional Laplacian is based on potential (electric field) theory, and not on reaction–diffusion theory. Thus, the appropriate case is *N* = 3 owing to the three-dimensional nature of the electric field associated with any charge distribution, regardless of their particular space distribution. Hence, our results also hold for one- and two-dimensional tissues, where a monopole/dipole charge distribution still generates a three-dimensional electrical field in the surrounding space, with the confined and outer components of this field subjected to different conductivities (or air permittivity). This is, in fact, the same principle underlying the computation of the pseudo-electrocardiogram signal [4,30] in any point outside the integration domain when using the standard cable equation.

However, this mathematical framework also holds with *N* = 2, as long as 0 < *α* < 2, and also in the case *N* = 1 under a simple modification [23]. For example, with *N* = 2 and *α* = 1, (2.4) and (2.5) lead to *C_*α*_* = 2*π* and *ϕ*(*r*) ∼ 1/*r*, which corresponds to the well-known dipole formulation in two spatial dimensions. As *α* approaches 2, there is a transition to the monopole corresponding to *ϕ*(*r*) ∼ log *r* and hence, as in the *N* = 3 case, the fractional model again represents a transition between the dipole and monopole distributions.

## 3. Role of tissue inhomogeneities on cardiac propagation

### 3.1. Conduction effects on myocardial depolarization

The depolarization of a cardiomyocyte is characterized by an initial deviation from its resting membrane potential, known as the AP foot, then followed by the rapid AP upstroke. Probably, the most comprehensive experimental study to date on the effects of tissue structure in this AP phase is still the work of Spach *et al.* [6], where the authors investigated the impact of wavefront propagation on the depolarization of canine cardiac tissue.

**Figure 2.**
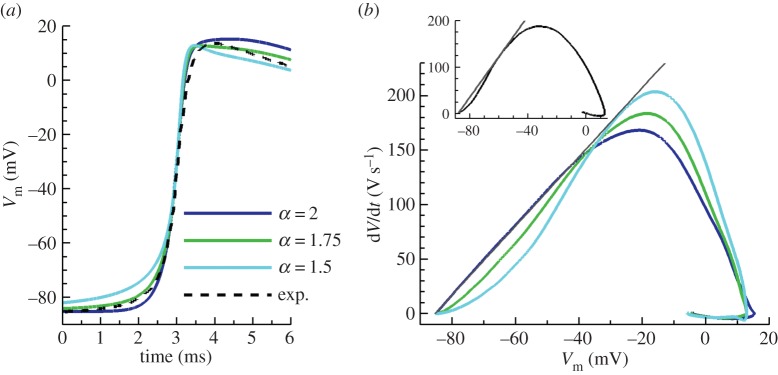
Effects of fractional diffusion on tissue depolarization. APs are recorded in the centre of a 4 cm cable in length. (*a*) Fractional propagation models (*α* < 2) significantly increase action potential foot width when compared with standard diffusion (*α* = 2). Experimental data (dashed line) illustrate a representative depolarization wavefront in healthy canine ventricular myocardium during longitudinal propagation. (*b*) Phase-plane trajectories during depolarization for the different values of the fractional power *α*, indicating a concave deviation from linearity in the fractional models as experimentally reported (inset). Experimental data adapted from [6]. (Online version in colour.)

Figure 2*a* illustrates the depolarization wavefront presented in [6] during longitudinal propagation (dashed line), compared with simulated waveforms using a biophysically detailed canine AP model [31]. Standard diffusion (*α* = 2) yields the narrowest AP foot, with increasing foot width for decreasing fractional powers. In particular, the value of *α* = 1.75 nicely replicates the observed experimental AP foot of this ventricular preparation, whereas standard diffusion underestimates its width and morphology. Furthermore, fractional diffusion induced only a small decrease in AP amplitude when compared with standard diffusion (≈2.4 mV for both *α* = 1.75 and *α* = 1.5).

Spach *et al.* further characterized the role of wavefront conduction in depolarization by analysing the *V*_m_ − d*V*_m_/d*t* phase-plane trajectories (figure 2*b*, inset). During longitudinal propagation, the majority of their ventricular and atrial impalements exhibited concave trajectories in the phase portrait (*n* = 40, 80%), indicating a deviation of the AP foot from exponential growth (i.e. a linear *V*_m_ − d*V*_m_/d*t* relationship). The rest of the preparations displayed mixed concave/convex trajectories, but all deviated from linearity. Figure 2*b* shows the phase-plane trajectories in the canine model obtained for different *α*. While standard diffusion produces a completely linear phase portrait (*α* = 2), fractional diffusion yields profiles with increasing degree of concavity for decreasing *α*. The mean experimental deviation of maximum d*V*_m_/d*t* from linearity was −15.1 V s^−1^ in ventricular muscle. This separation was quantified for the fractional diffusion models, resulting in −14.5 V s^−1^ for *α* = 1.75 and −34.9 V s^−1^ for *α* = 1.5. These results indicate that the depolarization of this particular experiment can be very well approximated by a fractional power close to *α* = 1.75. Moreover, although the actual range of deviation from linearity was not provided in their study, the authors classified their ventricular impalements as exhibiting ‘minor’, ‘moderate’ and ‘considerable’ concavity degrees (respectively, 9%, 27% and 64% of ventricular preparations, *n* = 22). This classification suggests that fractional powers *α* < 1.75 (resulting in more pronounced concavities) can also be viable in healthy myocardium. In this regard, newborn tissues, richer in microvasculature and discontinuities, exhibited even larger degrees of concavity than adult myocardium [6].

**Figure 3.**
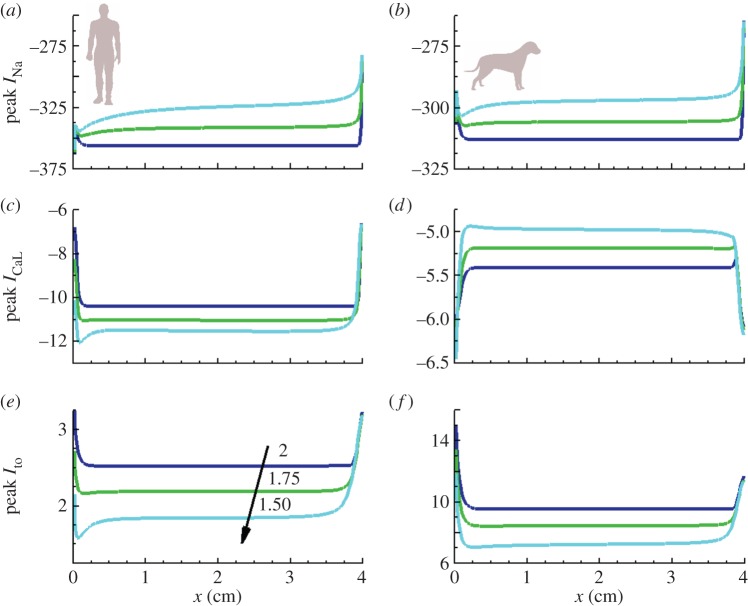
Peak value distributions (*μ*A/*μ*F) for predominant currents during depolarization. Results are presented for human (*a,c,e*) and canine (*b,d,f*) cell models, measured in a tissue cable of 4 cm length stimulated at its boundary. Despite displaying same magnitudes at the distal boundary, fractional propagation models (*α* < 2) significantly increase the range of influence of this region compared with standard diffusion (*α* = 2). (Online version in colour.)

Peak value distributions for the three principal currents during depolarization are depicted in figure 3 for human [32] and canine [31] cell models. Despite model-specific magnitudes, almost constant profiles are found for all currents in the case of standard diffusion (*α* = 2), only influenced by the stimulus and distal boundary sites. However, the effects of fractional diffusion on the AP foot yield a wider range of influence for these regions. Peak magnitudes of the fast sodium, *I*_Na_, and the transient outward current, *I*_to_, were reduced in both cellular models, thus leaving upstroke amplitude almost unaffected through the tissue. These results were also consistent with those in the description of rabbit electrophysiology [33]. Conversely, the behaviour of the L-type calcium current, *I*_CaL_, was model-dependent, exhibiting a modest decrease in dog, whereas a small increase in human and rabbit models. No significant changes were observed in the rest of transmembrane currents.

### 3.2. The inverse AT–APD relationship

**Figure 4.**
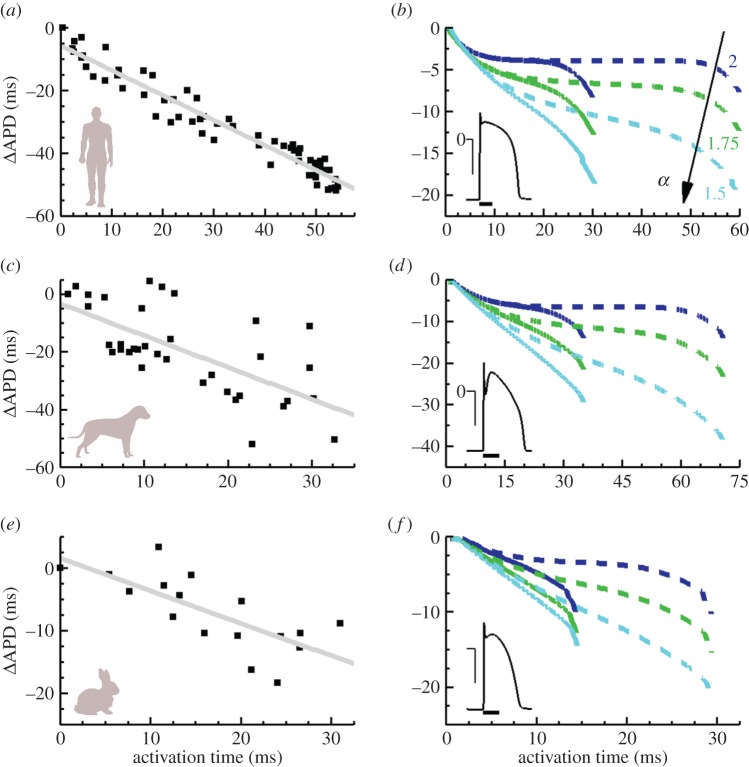
Inverse AT–APD relationship in ventricular tissue. Left: experimental AT–APD relationships in human (*a*), canine (*c*) and rabbit (*e*) cardiac tissue (adapted from [34–36]). Linear regression fits indicate an inverse correlation between both magnitudes. Right: fractional effects in APD dispersion for human (*b*), dog (*d*) and rabbit (*f*) cell models. Colour indicates fractional derivative order. Solid and dashed lines denote shorter and longer tissue cables (2 and 4 cm length, halved for rabbit simulations), stimulated at their left boundary (2 × threshold; 2 ms duration; 1 Hz pacing for human and canine, 2 Hz for rabbit). APD dispersion is substantially larger in the fractional diffusion models, closer in magnitude and morphology to the experimental values. Insets show single-cell action potentials for the different cell models (horizontal scale bars, 100 ms; vertical bars, 50 mV). (Online version in colour.)

A compelling mechanism of the intact heart, reported in multiple studies and different species, is the shortening of AP duration (APD) during propagation, also known as the inverse AT–APD relationship. To better illustrate this aspect, the left column of figure 4 provides representative experimental data for *in vivo* human [34], dog [35] and isolated rabbit hearts [36].

The contribution of tissue inhomogeneities, as modelled by fractional diffusion, to APD dispersion (ΔAPD) was investigated in tissue cables for biophysically detailed models of human [32], dog [31] and rabbit [33], as shown in the right column of figure 4. Standard diffusion (*α* = 2) yields moderate ΔAPD values, regardless of cell type. More remarkable is the fact that, for all cell models, ΔAPD distributions turn into nearly constant profiles once the domain size becomes comparable to the AP wavelength [37]. On the other hand, ΔAPD increasingly grows for decreasing fractional power, more closely resembling the ΔAPD profiles reported experimentally.

**Figure 5.**
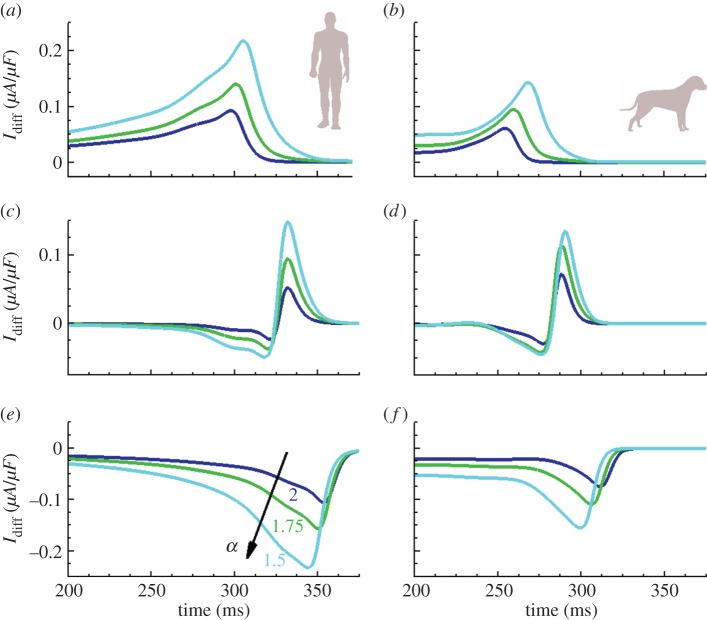
Diffusive repolarization currents for human and canine cell models. Results are presented at proximal (*a*,*b*), intermediate (*c*,*d*) and distal (*e*,*f*) locations from stimulus site, measured in a tissue cable of 4 cm length stimulated at its boundary. Coupling currents are substantially increased at all sites for fractional models (*α* < 2) compared with standard diffusion (*α* = 2). (Online version in colour.)

In the absence of regional gradients in the expression of ionic currents, the main mechanism contributing to APD dispersion in cardiac tissue is cell-to-cell electrotonic coupling. As elegantly discussed in [37], the electrotonic current is large and positive within the stimulated region (figure 5*a,b*), which lengthens APD, whereas it is large and negative at the boundaries (figure 5*e*,*f*), thus contributing to APD shortening at distal locations of the tissue. As illustrated in figure 5 for the human and canine cellular models, cell-to-cell electrotonic load during repolarization is substantially larger at all sites for fractional compared with standard diffusion, hence amplifying tissue coupling effects on APD dispersion. Electrotonic currents are also larger for cell models with a sharper repolarization phase (see individual APs in figure 4), in agreement with previous results [37]. Despite specific AP morphology, the influence of fractional diffusion in increasing repolarization effects was consistent for all the studied cell models.

### 3.3. The modulated dispersion of repolarization

Another important characteristic of cardiac tissue, owing to its implications in arrhythmogenesis, is the nonlinear response referred to as APD restitution. Among existing protocols, the most clinically relevant is the standard or S_1_–S_2_ restitution. For steady-state conditions at a fixed S_1_ pacing cycle length, this protocol relates APD at any tissue point as a function of their preceding diastolic interval, APD*^n^* = *f*(DI*^n^* ^−^^1^), under a premature S_2_ stimulus. Here, DI*^n^* ^−^^1^ = CI–APD*^n^* *^−^* ^1^ and CI is the coupling interval (time difference between S_1_ and S_2_ stimulations), whereas superscripts refer to the beat number.

**Figure 6.**
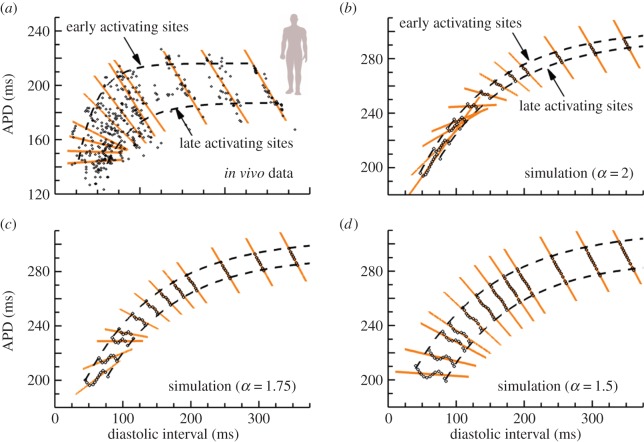
Global DI–APD relationship changing with premature coupling interval in a standard restitution protocol. (*a*) Experimental data from multiple recording sites from healthy human endocardium (adapted from [34]). Linear regression lines are shown for APD versus DI at each test coupling interval, exhibiting a progressive flattening of slope as the coupling interval shortens. (*b*) Global DI–APD dependence in a simulated cable of human cardiac tissue of 4 cm length using standard diffusion (*α* = 2). Not only is dispersion between early- and late-APD restitution curves small, but regression lines manifest a rapid inversion of slopes at short coupling intervals. (*c*,*d*) Global DI–APD dependence for fractional diffusion models (*α* = 1.75 and *α* = 1.5, respectively). The separation between early- and late-APD restitution curves increases for decreasing fractional order *α*, also recovering the progressive flattening of regression lines. (Online version in colour.)

Owing to the shortening of APD during propagation, a range of restitution profiles may also exist along the path of activation. Such an effect in APD restitution has been reported in human [34] and animal [38,39] studies. Experimental evidence for one patient with healthy ventricles is presented in figure 6*a*. For each coupling interval, local APDs from numerous ventricular sites are plotted against their preceding DIs, and a local regression line is drawn. For test beats close to the basic cycle length (figure 6*b*), the regression line has a slope of −1. As the coupling interval is shortened, DIs decreased, and the restitution curve acted to reduce APD dispersion. This results in the progressive flattening of regression lines known as modulated dispersion of repolarization, with electrode sites having shorter DIs exhibiting a larger APD reduction compared with electrodes sites with longer DIs [34].

The ability of the different propagation models in reproducing the modulated dispersion of repolarization was inspected for human electrophysiology [32]. Figure 6*b* shows results for standard diffusion (*α* = 2). The APD difference between early and late activating sites is small in this case, owing to the minimum role of standard diffusion in the inverse AT–APD relationship. More intriguing is the rapid inversion of DI–APD regression lines at medium and short coupling intervals, not observed in the *in vivo* data. Results for fractional diffusion models are also presented, for *α* = 1.75 (figure 6*c*) and *α* = 1.5 (figure 6*d*). As the fractional order *α* is decreased, not only does the APD difference between early and late activating sites increase, but the progressive flattening of regression lines is also recovered. Two factors are involved in the recovery of this gradual flattening. First, the APD decreases along the activation pathway, and, second, there is an increased dispersion of local DIs in the tissue, as can be observed by comparison of figure 6*b–d*. Both factors are interrelated, because DI*^n^* ^−^^1^ = CI − APD*^n^*^−^^1^. Thus, the larger the APD dispersion in the basic beat, the bigger the resulting dispersion of DIs preceding the premature stimulus.

**Figure 7.**
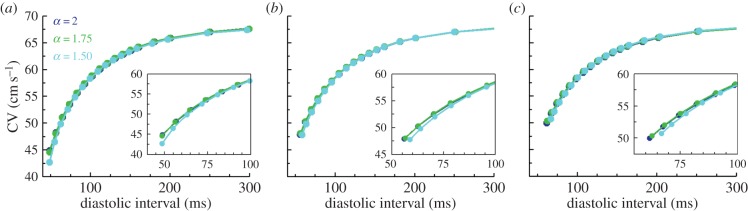
Conduction velocity restitution profiles in a human model of cardiac electrophysiology. CV is measured at proximal (*a*), medial (*b*) and distal (*c*) locations in a tissue cable of 4 cm length. Recording sites are, respectively, located at 1, 2 and 3 cm from the stimulated boundary. Insets provide details of CV restitution at short diastolic intervals, whereas colour indicates derivative order. (Online version in colour.)

An additional property known to interact with APD restitution in the modulation of APD patterns is conduction velocity (CV) restitution [40,41]. Equivalent to APD restitution, this relates CV as a function of their preceding DIs, CV*^n^* = *f*(DI*^n^* ^−^^1^). Fractional diffusion effects on CV restitution are investigated in figure 7. Only slight modifications in CV restitution profiles are observed at short DIs for decreasing *α*, owing to the increased dispersion of local DIs for the fractional diffusion models. Therefore, fractional diffusion allows the reproduction of key properties in the dispersion of repolarization in cardiac tissue, without altering other important properties of cardiac conduction.

## 4. Discussion

The new modelling framework presented in this contribution aims to probe mathematical descriptions of cardiac tissue with the macroscopic effects of structural heterogeneity on impulse propagation. Our findings, analysed in cellular models of human, dog and rabbit electrophysiology, indicate that the secondary electrical sources created by tissue inhomogeneities (as modelled by fractional diffusion) play a significant role in explaining a number of relevant characteristics observed during myocardial excitation. These include conduction effects on the AP foot during depolarization, the inverse AT–APD relationship, and the modulated dispersion of repolarization. Furthermore, the resulting approach provides a unified framework that allows for the joint interpretation of all these factors, solely based on the intrinsic heterogeneous nature of cardiac tissue. In fact, both clinical, experimental and theoretical studies have previously suggested that structural heterogeneity may actively modulate the course of impulse propagation and recovery of excitability in cardiac tissue [6,15,42]. However, limitations of conventional modelling techniques hamper our ability to provide novel insights into the influence of tissue microstructure in these regards.

Shortening of APD along the activation path has been reported in human and different animal species [34–36,38]. Importantly, this inverse AT–APD relationship is considered a natural protective mechanism of the intact heart [34], because, as APD shortens, so does dispersion of repolarization, which is widely accepted as being arrhythmogenic [35,43]. However, this property of wavefront propagation is not accurately reproduced by standard cable equation models of cardiac tissue, which yield almost entirely constant AT–APD distributions (figure 4). Our results suggest that tissue inhomogeneities assert a crucial role in the mode of action of electrotonic current flow, thus explaining the inverse AT–APD coupling and highlighting its implications as the underlying mechanism regulating the modulated dispersion of repolarization. They also indicate a tissue modulation of ionic currents acting during AP depolarization. Moreover, experimental evidence indicating a structural role of the tissue on membrane currents and on the morphology of the initial part of the depolarizing phase has been also reported [6,15].

On the other hand, several guinea pig studies have suggested that regional differences in the expression of ionic currents may underlie APD shortening during propagation [38,39]. Whereas we cannot exclude their possible contribution to total APD dispersion, or the combined effect of both factors, other experimental studies have shown electrotonic modulation of APD dominates the effects of intrinsic differences in cellular repolarization characteristics [44]. Although the main goal of this work was to characterize how tissue microstructure influences electrical function in an otherwise homogeneous condition, it will be interesting to analyse how fractional diffusion modulates existing ionic gradients in cardiac tissue, and their contribution, for instance, to the body-surface electrocardiogram.

In the past, traditional approaches to understand the role of tissue heterogeneity in cardiac conduction have been based on the combination of standard diffusion models with high resolution anatomical reconstructions of tissue structure. The level of anatomical detail obviously depends on mesh resolution, and current discretizations suffice to capture from localized fibrotic patches [42] to medium-sized vessels [45,46]. Finer anatomical features, such as capillaries or intercellular cleft spaces, would imply, however, the use of anatomical models at submicrometre resolution (figure 1), which are currently intractable even with the most advanced high-performance facilities. Some novel methods have been recently proposed to overcome some of these limitations, such as the use of discontinuous finite-elements to represent fibrotic clefts [47]. Furthermore, measuring the heterogeneity at these microscales and then estimating appropriate conductances imposes additional difficult challenges. As an alternative, the proposed fractional diffusion models represent a flexible approach to characterize the role of cardiac microstructure in electrical propagation in terms of computational tractability, because spatial discretization is retained at a mesoscopic and not subcellular scale. However, their numerical resolution can impose a number of constraints when compared with standard diffusion, because the fractional Laplacian yields full, instead of sparse, matrices. Nevertheless, new efficient techniques, that avoid the explicit calculation of the fractional operator, have been recently proposed for these types of systems [48,49]. In particular, and for sufficiently regular geometries, the methods presented in [49] achieve the same computational cost as associated with standard diffusion.

Therefore, fractional diffusion models may have potential implications in advancing our understanding on the mechanisms of dispersion of repolarization and its modulation by premature beats. Our findings indicate that fractional powers *α* < 2 reproduce many interesting tissue properties in a variety of human and animal cellular models. Although we have concentrated our analysis in the upper part of its allowable range, lower values of *α* are nevertheless admissible, and a rigorous methodology needs to be developed to properly estimate these values. Different imaging modalities have been recently proposed to characterize fractional diffusion transport in neural tissue [17,50], and they might be extended as well for their application to cardiac tissue. Importantly, we are not suggesting that there should be a unique value to represent heterogeneities. Rather, we suggest that there are ranges of suitable values of *α* in different settings (such as healthy or diseased states), and this is consistent with important new modelling approaches centred on the concept of populations of models to represent biological variability [51–53]. Indeed, although a constant value of *α* is associated with the average level of tissue inhomogeneity that is spatially distributed, more localized inhomogeneities (such as the epicardial layer being richer in vasculature than the endocardium) can be considered through space-varying fractional powers, whereas larger anatomical defects (such as main blood vessels) could still be incorporated in the mesh generation process. All these points will be addressed in future work.

## 5. Methods

### 5.1. Models and simulations

Simulations were conducted in one-dimensional fibres of cardiac tissue of length as specified in the main text, using AP models of canine [31], human [32] and rabbit [33] ventricular electrophysiology. All models provide biophysically detailed descriptions of the main transarcolemmal currents, calcium handling and ion homeostasis in the considered species.

At the tissue level, macroscopic properties such as CV must be captured by the specific propagation model, regardless of its mathematical description. The diffusion coefficient, *D_*α*_*, in equation (1.1) was thus adjusted for the fractional models to match the CV in standard diffusion (*α* = 2), as measured in the centre of tissue cables of 2 cm length. For standard diffusion, diffusion coefficients of 1.2, 1.0 and 1.4 cm^2^ s^−1^ were used for human, dog and rabbit models, respectively, to yield a CV of 70, 58 and 67 cm s^−1^, as experimentally reported.

### 5.2. Protocols for validation against experimental data

Tissue models were initialized with single-cell steady-state conditions at the specified cycle lengths, and paced as indicated in the main text until the relative difference in ΔAPD was less than 0.5% in two consecutive heart beats. Activation time was determined at the steepest upstroke of the AP, whereas repolarization time was quantified at 90% of repolarization, matching reported experimental conditions. APD was measured as the difference between the repolarization and activation times. Dispersion in any of these values was measured as the difference between the maximum and minimum values obtained over the entire domain.

APD restitution curves were calculated in one-dimensional cables of 4 cm length. The cable was paced until steady-state at one end with a stimulus of strength 2 × diastolic threshold and a cycle length of 1000 ms, then introducing test pulses over a range of different coupling intervals. The resulting DI–APD pairs were computed for all points in the tissue.

### 5.3. Numerical techniques

All models were integrated with a temporal resolution of Δ*t* = 0.0025 ms, with spatial discretization of Δ*x* = 1/64 cm ≈ 150 μm. Simulations were performed using a semi-implicit Fourier spectral method as described in [49,54], with non-flux boundary conditions to ensure conservation of charge. In brief, given a complete set of orthonormal eigenfunctions {*φ_j_*} for the Laplacian satisfying the boundary conditions in the interval of length *L*, with corresponding eigenvalues *λ_j_*, i.e. (−Δ)*φ_j_* = *λ*_j_*φ_j_*, then the fractional Laplacian is given by5.1
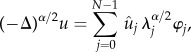
where *N* is the number of discretization points, and coefficients 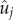 are computed by the discrete cosine transform. Using a backward Euler stencil for the time derivative, and after rearrangement of terms, the time–space discretization for the *j*th Fourier mode of equation (1.1) simply becomes5.2
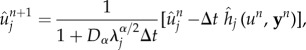
where *λ_j_* = (*j*π*/L*)^2^, and *u* ≡ *V*_m_, *h*(*u*, **y**) = 1*/C*_m_ (*I*_ion_(*u*, **y**) − *I*_stim_) have been used to simplify notation. The remainder of state variables **y** is updated using an explicit Euler scheme.

## Data accessibility

All cellular models representing the species-dependent ionic terms were coded using their curated implementations in the CellML repository (http://models.cellml.org/electrophysiology).

## Appendix A. Multi-scale modelling in heterogeneous media

Here, we briefly discuss different approaches for multi-scale modelling in the presence of heterogeneity.

Diffusion in heterogeneous media can sometimes be well approximated by a homogeneous standard diffusion medium whose diffusive properties are close to that of the real medium. The key to this homogenization is the nature of the spatial scales in the underlying heterogeneous media. It is this idea that underpins the bidomain model. In its simplest form, homogenization assumes the domain is defined at a macroscopic scale, *L*, whereas the characteristic length of the heterogeneities defines a microscopic scale, . Given a conductivity tensor *σ*(*x*/*ɛ*), homogenization studies the solution of the underlying equations as *ɛ* → 0 (i.e. as heterogeneities become vanishingly small), aiming to replace the rapidly oscillating coefficients *σ*(*x*/*ɛ*) by an effective domain characterized by constant coefficients, . It is possible to apply these ideas to multiple scales and layered domains, but homogenization becomes increasingly difficult [55] and assumptions have to be made about the regularity of *σ*. However, when the number of scales becomes large, without clear separation, homogenization fails.

A classic example in potential theory, going back to the work of Maxwell and Rayleigh and the so-called Maxwell–Claussius–Mossotti formula, is the study of the effective electrical properties of a large sphere of radius *R* with conductivity *D*_1_ and a number *N* of spherical inclusions of radius *δ*, with conductivity *D*_2_. Then, the Maxwell–Claussius–Mossotti formula allows for an effective approximate conductivity for the whole domain, but only in the case when the relative volume . For more heterogeneous situations, when either the size or the number of inclusions increases and the relative volume becomes , homogenization by a traditional approach is not possible, and new approaches are needed [56].

Some recent work on modelling heterogeneity has considered the behaviour of diffusive particles in random fields through stochastic differential equations. The fundamental setting is that of an Itô process driven by additive Wiener noise, *w_t_*, of the form

A number of authors [20–22] have shown that a superdiffusive behaviour can arise from the above diffusive process if *D*(**x**) has a large number of spatial scales that are not well separated (a characteristic of heterogeneity). Essentially, this is based on considering expansions of the form  where *h_n_*(**x**) are smooth functions of period one, appropriately rescaled in width and amplitude by *R_n_* and *γ_n_*, respectively. For example, if *γ_n_* = *γ^n^* and *R_n_* = *ρ^n^*, then the width of the ensemble increases as *t*^(1 +^ *^q^*^)/2^, *q* = log *γ*/log *ρ*, in contrast to the normal *t*^1/2^ case.

An alternative approach for the characterization of transport processes in fields that are non-uniform on multiple scales is the use of multi-scale diffusivities. The first model of this type is attributed to Richardson in the mid-1920s [57], for the study of the diffusion of particles in turbulent flows including vortices whose size is commensurable to the distance between particles. The proposed semi-empirical diffusion equation is

A 1

where  and coefficient *γ* = 4/3, which was later confirmed theoretically by Kolmogorov [58]. In fact, the physical meaning of  is simply that of a continuous diffusive process at all possible space scales. Solutions to the Richardson model with *γ* = 4/3 show a superdiffusive process where the width of a packet increases as *t*^3/2^, and the distribution of a random vector proves to be non-Gaussian at all scales. The above results have been generalized for arbitrary *γ* in a very recent paper [59], showing that if , then the width of a packet scales as *t*^1/(2 −*γ*)^, so that if , then superdiffusivity arises, whereas *γ* < 0 yields subdiffusion.

Monin [60] developed an alternative model to (A 1) by considering a diffusion operator of the form

Application of the inverse Fourier transformation leads to the following equation with a fractional Laplacian for the density

A 2

where *α* = 2/3. The analytic solution to this equation can be obtained in terms of fractional stable radial densities [61], exhibiting the same *t*^3/2^ superdiffusive process than the original Richardson model (A 1), and as *t*^1/*α*^ for arbitrary *α*. Note at this point the equivalence between the multi-scale model written in the form of (A 1) and the fractional Laplacian representation given by equation (A 2), which underlies our fractional approach to describe cardiac tissue.

The important message here is that, when heterogeneity is manifested as a very large number of scales that cannot be separated, superdiffusion can arise from purely diffusive processes. However, the characterization of these processes is one of the very hard problems in statistical physics, and general theories are difficult to construct. Nevertheless, it is clear that superdiffusion can arise in natural ways, and such characterizations will rely on a combination of theory, simulation and experiments.
